# Umbilical Cord Mesenchymal Stem Cells in Amyotrophic Lateral Sclerosis: an Original Study

**DOI:** 10.1007/s12015-020-10016-7

**Published:** 2020-07-28

**Authors:** Monika Barczewska, Stanisław Maksymowicz, Izabela Zdolińska-Malinowska, Tomasz Siwek, Mariusz Grudniak

**Affiliations:** 1 Department of Neurosurgery, University of Warmia and Mazury, Olsztyn, Poland; 2 Instytut Terapii Komórkowych S.A., FamiCord Group, Olsztyn, Poland; 3 University Clinical Hospital, Olsztyn, Poland; 4 Department of Psychology and Sociology of Health and Public Health, Collegium Medicum, University of Warmia and Mazury, Warszawska 30, 10-082 Olsztyn, Poland; 5 Polski Bank Komórek Macierzystych S.A., FamiCord Group, Warsaw, Poland; 6 Department of Neurology, University of Warmia and Mazury, Olsztyn, Poland

**Keywords:** Amyotrophic lateral sclerosis, WJ-MSC, ALS, Umbilical cord, Survival, Progression rate, Medical experiment

## Abstract

**Objective:**

Amyotrophic lateral sclerosis (ALS) is still incurable. Although different therapies can affect the health and survival of patients. Our aim is to evaluate the effect of umbilical mesenchymal stem cells administrated intrathecally to patients with amyotrophic lateral sclerosis on disability development and survival.

**Methods:**

This case-control study involved 67 patients treated with Wharton’s jelly mesenchymal stem cells (WJ-MSC). The treated patients were paired with 67 reference patients from the PRO-ACT database which contains patient records from 23 ALS clinical studies (phase 2/3). Patients in the treatment and reference groups were fully matched in terms of race, sex, onset of symptoms (bulbar/spinal), FT9 disease stage at the beginning of therapy and concomitant amyotrophic lateral sclerosis medications. Progression rates prior to treatment varied within a range of ± 0.5 points. All patients received three intrathecal injections of Wharton’s jelly-derived mesenchymal stem cells every two months at a dose of 30 × 10^6^ cells. Patients were assessed using the ALSFRS-R scale. Survival times were followed-up until March 2020.

**Results:**

Median survival time increased two-fold in all groups. In terms of progression, three response types measured in ALSFRS-R were observed: decreased progression rate (*n* = 21, 31.3%), no change in progression rate (*n* = 33, 49.3%) and increased progression rate (*n* = 13, 19.4%). Risk-benefit ratios were favorable in all groups. No serious adverse drug reactions were observed.

**Interpretation:**

Wharton’s jelly-derived mesenchymal stem cells therapy is safe and effective in some ALS patients, regardless of the clinical features and demographic factors excluding sex. The female sex and a good therapeutic response to the first administration are significant predictors of efficacy following further administrations.

**Graphical Abstract:**
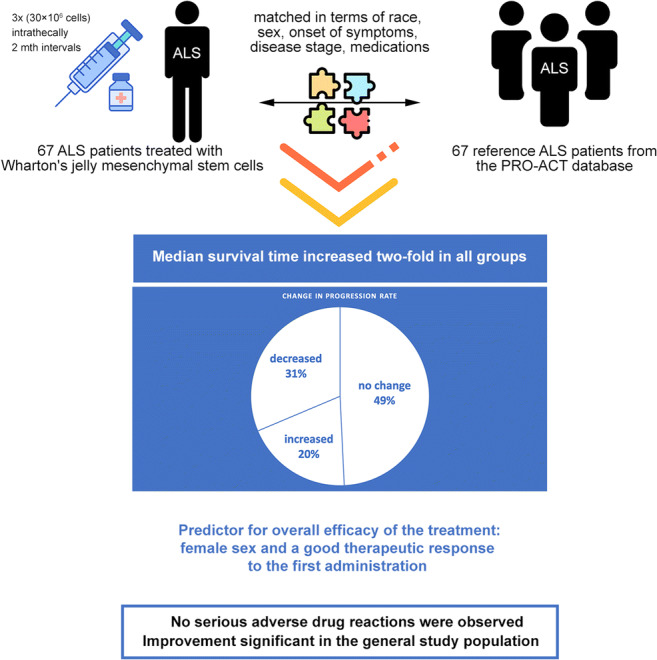
Medical therapeutic experiment with retrospective case-control analyses

**Electronic supplementary material:**

The online version of this article (10.1007/s12015-020-10016-7) contains supplementary material, which is available to authorized users.

## Introduction

There is no causal therapy for amyotrophic lateral sclerosis (ALS) – a rare, fatal, progressive disease that has been rapidly increasing [1] and occurs de novo in 90% [2]. The studies using iPSC-derived motor neurons revealed the complicated molecular nature of this disease related to mitochondrial dysfunction and ER stress, NF aggregate formation, hyperexcitability, and channel deficits [3]. There is still only one approved drug in Europe – riluzole, which extends survival by 3 months on average. No new drugs have been registered for this indication since 1994 [4], partly due to our incomplete understanding of the complex pathogenesis of motor neuron degeneration [5]. Recently, another drug, edaravone, has been registered in Japan and the USA. There is therefore still a huge unmet need for a treatment that could effectively alter the progression rate of this debilitating disease.

Although it is well known that MSC differentiate into neural cells and neuroglia [6], over the course of the last several years stem/stromal cell therapy was proposed for treating ALS based on small observational studies. Evidence from preclinical studies conducted with the animal ALS (SOD1) model provided promising results and led to the initiation of some clinical studies^-^ [7–9], Previously, the safety and efficacy of mesenchymal stem cells (MSC) administration was described in small studies which did not evaluate overall survival. The aim of our study was to evaluate the effect of compassionate use of Wharton’s jelly-derived mesenchymal stem cells (WJ-MSC) in terms of progression and survival in ALS patients. This was done in the form of a medical therapeutic experiment (described elsewhere [10]) with retrospective case-control analyses.

## Materials and Methods

### Patients

The study was conducted at Instytut Terapii Komórkowych S.A. in Olsztyn, Poland (Cell Therapies Institute, FamiCord Group) in cooperation with the Departments of Neurology and Neurosurgery, School of Medicine, Collegium Medicum of the University of Warmia and Mazury in Olsztyn, Poland. The treatment was reviewed by the Ethics Committee of the School of Medicine, University of Warmia and Mazury in Olsztyn, Poland and performed in accordance with the *Declaration of Helsinki*. Prior to starting the experiment, all patients signed an informed consent form. Patients enrolled into the study were recruited between 2015 and 2018. Enrolled patients met the following profile: clinical diagnosis of definite ALS based on the El Escorial World Federation of Neurology criteria [11], ventilatory independence, age 20–78 years and the ability to attend clinic visits alone or with support. The following laboratory tests were mandatory: C-reactive protein (CRP), sodium, potassium, glucose, blood cell count, coagulation, urea and creatinine.

### MSCs Harvesting and Processing

WJ-MSCs were donated from healthy newborns following maternal qualification based on a medical questionnaire conducted after the parents signed an informed consent form. The harvested umbilical cords were transported to the laboratory under monitored conditions and processed within 48 h of delivery. Once disinfected, they were dissected, stripped of any blood vessels and minced into 2 cm^3^ pieces, which were placed into 6-well plates. The whole procedure was conducted without the use of enzymes. Tissue explants were cultured and incubated at 37 °C in 5% CO_2_ in air. After 1–2 h, nonadherent cells were washed off and any adherent cells were further expanded. The tissue explants were removed after 2–3 weeks of culture. When the adherent cells reached 90% confluence, they were passaged and reseeded for further expansion at 1.2 × 10^4^ cells/cm^2^ a 75 cm^2^ tissue culture flask (BD). To evaluate their numbers, cells were detached and counted in a hemocytometer. After reaching a sufficient number of cells, they were immunophenotyped, cryopreserved and stored in liquid nitrogen vapor.

The cells fulfilled the criteria described by Dominici et al. [12] Briefly, the cells were detached from the plate using a trypsin solution upon reaching 60–80% confluence. In preparation for flow cytometry, they were incubated (30 min in the dark) with fluorochrome-conjugated antibodies against MSC-negative surface markers (CD34 FITC, CD14 FITC, CD19 FITC, CD45 FITC and HLA-DR FITC) and MSC-positive surface markers (CD73 PE, CD90 PE, CD105 PE and HLA ABC FITC). Next, the cells were washed, resuspended in Cell Fix solution, and analyzed in a BD FACSCalibur cytometer. Murine anti-IgG1 FITC and anti-IgG1 PE were used as controls. Before administration to the patients, the cells were thawed in a 37 °C water bath. Cell viability was determined with the trypan blue dye exclusion test, based on a thawed reference sample. We did not monitor the proliferation rate or any other indicator of cellular senescence. Patients received only early-passage cells (at the fifth passage or less). The unit volume, number, vitality and morphology of the cells, microbiological purity, results of the serological tests, absence of endotoxins, and immunophenotype of the cells in the finished product adhered to the specifications of the Chief Pharmaceutical Inspectorate.

### Reference Group

Reference subjects were obtained from the PRO-ACT database (www.nctu.partners.org/proact). This database contains more than 10,000 patient records from 23 different clinical studies (phase 2/3) and it has been used earlier also by another authors as a reference data set [13, 14]. Each patient was paired with a reference subject, fully matched in terms of race, sex, disease stage (assessed with the FT9 scale), treatment, onset symptoms (bulbar/spinal). Patients with fast progression were identified based on an ALSFRS-R score Loss of >1 point per month and slow progression was defined as <1 point/month [15]. Age differences in 52 of 67 patients (77.6%) were within ± 5 years and in 63 of 67 (94.0%) were within ± 10 years (Supplementary Fig. 1). In terms of progression rates 65 of 67 Pairs (97.0%) were matched within the range ± 0.3 point/month and no patient exceeded 0.5 point/month (Table 1). In 45 patients (67.2%) the difference equaled zero (Supplementary Fig. 2)

**Table 1:** Patient characteristics (all differences were not significant in chi-square or Mann-Whitney test)

	Treatment group *N* = 67	Reference group *N* = 67
Sex		
Men	37 (55.2%)	37 (55.2%)
Women	30 (44.8%)	30 (44.8%)
Age, years		
median (range)	57 (36–78)	58 (31–76)
[interquartile range]	[53–63]	[53–66]
Riluzole		
yes	50 (74.6%)	50 (74.6%)
no	17 (25.4%)	17 (25.4%)
Onset		
Spinal	51 (67.1%)	51 (67.1%)
Bulbar	16 (23.9%)	16 (23.9%)
Stage at baseline		
Less advanced (FT 9 stage 1 or 2)	46 (66.7%)	46 (66.7%)
More advanced (Ft9 stage 3, 4 or 5)	21 (31.1%)	21 (31.1%)
Progression		
fast (> = 1 point/month)	26 (38.8%)	26 (38.8%)
slow (<1 point/month)	41 (61.2%)	41 (61.2%)
Progression [point/month]		
median (range)	−0.7 (−4.2 to 0.0)	−0.7 (−2.2 to 0.0)
[interquartile range]	[−1.4 to −0.3]	[−1.3 to −0.3]

### ALSFRS-R and Fine’til 9 Scales

ALSFRS-R is the revised (R) functional rating scale for patients with ALS. The basic version assesses the functions of speech, swallowing, self-care and patient mobility [16, 17]. There are 12 questions in the scale, with separate additional items concerning cutting food (for patients with or without gastrostomy). Answers correspond to scores from 0 to 4, where 0 is the largest possible deficit in a given area (e.g. loss of useful speech) and 4 corresponds to lack of any deficit (e.g. normal speech processes). The total score ranges from 0 to 48 points.

FT9 is based on the ALSFRS-R and was described as a tool enabling stratification of patients at various ALS stages [18]. This approach defines 5 disease stages - from 1 to 5 (FT9–1 to FT9–5). Stage 1 means the initial stage (minimally involved) and 5 denotes the most advanced stage of the disease. There are 4 main domains in the ALSFRS-R score (bulbar, upper limb, lower limb, breathing). All 4 domains include 3 separate questions, with a maximum of 4 points to be assigned for each and a maximum total of 12 points for each domain. If a patient receives less than 9 points in one of the 4 main domains, they are staged as FT9–2, and if 2 domains receive less than 9 points – as FT9–3 etc. Patients staged as FT9–1 have not received less than 9 points total in any of the ALSFRS-R domains.

### Administration Procedure

MSCs were administered intrathecally. Following a typical lumbar puncture, 2 mL of CSF was collected and the same volume of WJ-MSC suspension in 1 mL 5% human albumin was administered via a needle inserted at the L3/L4 level with the patient in the supine or sitting position. Patients received three MSCs administrations at 2-month intervals, following the same procedure each time. All patients received a WJ-MSC dose of 30 × 10^6^ cells at each injection.

### Clinical Evaluation

Clinical evaluation during the study involved physical and neurological examination at each MSCs administration visit. ASLFRS-R assessments were also performed at each administration visit. Additionally, to assess the rate of disability progression, a retrospective ALSFRS-R clinical assessment was performed, covering the six months prior to study entry. The average monthly ASLFRS score was calculated for each patient separately for the pre-treatment and treatment periods. Upon enrollment all patients were stratified according to FT9 level.

Decreased progression rate was defined as a difference of >3 points in the ALSFRS-R between treated vs. reference patients. No change in progression rate (lack of response) was defined as a difference within <3 points and > −3 in the ALSFRS-R. Increased progression rate was defined as a difference of <−3 points in the ALSFRS-R.

### Survival Time Analysis

Survival was assessed since the enrollment. Data were obtained by the investigator via telephone and e-mail. At times the patient’s families informed the investigators themselves about the patient’s death. The last survival data update is from March 2020. Statistical methods used for survival analysis are described in the next paragraph.

### Statistical Analysis

Patients who received at least three MSCs injections and were assessed at each administration visit were included into this analysis. In addition, the average monthly score decrease rate for the period before and during treatment was calculated. Microsoft Excel and Statistica 13 (TIBCO Software Inc.) were used to perform the analysis. To assess the significance of the difference between the predicted and observed values, the Wilcoxon signed-rank test was used. The association between categorized progression rates (fast/slow) and sex or ALS type was assed using the chi-square test. Survival analysis was performed using a Cox proportional hazard ratio and Kaplan-Meyer curves compared in log-rank and Cox-Mantel tests. In addition, linear and logistic regression, as well as discriminant analysis were performed to identify clinical response type predictive factors. In all cases, 0.05 was defined as the significance level. Statistical analysis was performed in the whole study population as well as in subgroups categorized on the basis of the clinical response and initial clinical and demographic characteristics. The power of the survival analysis indicated that difference in survival rate that we observed at the end of the observation (approx. 40% vs 0%) allows to achieve 90% statistical power in the sample size 11 person per one group (22 persons in the whole study population). In case of 67 patients per group, the statistical power for such proportions equals 100% (Supplementary Fig. 3).

## Results

### MSCs Treatment Extends Survival in ALS Patients

**Fig. 1:**
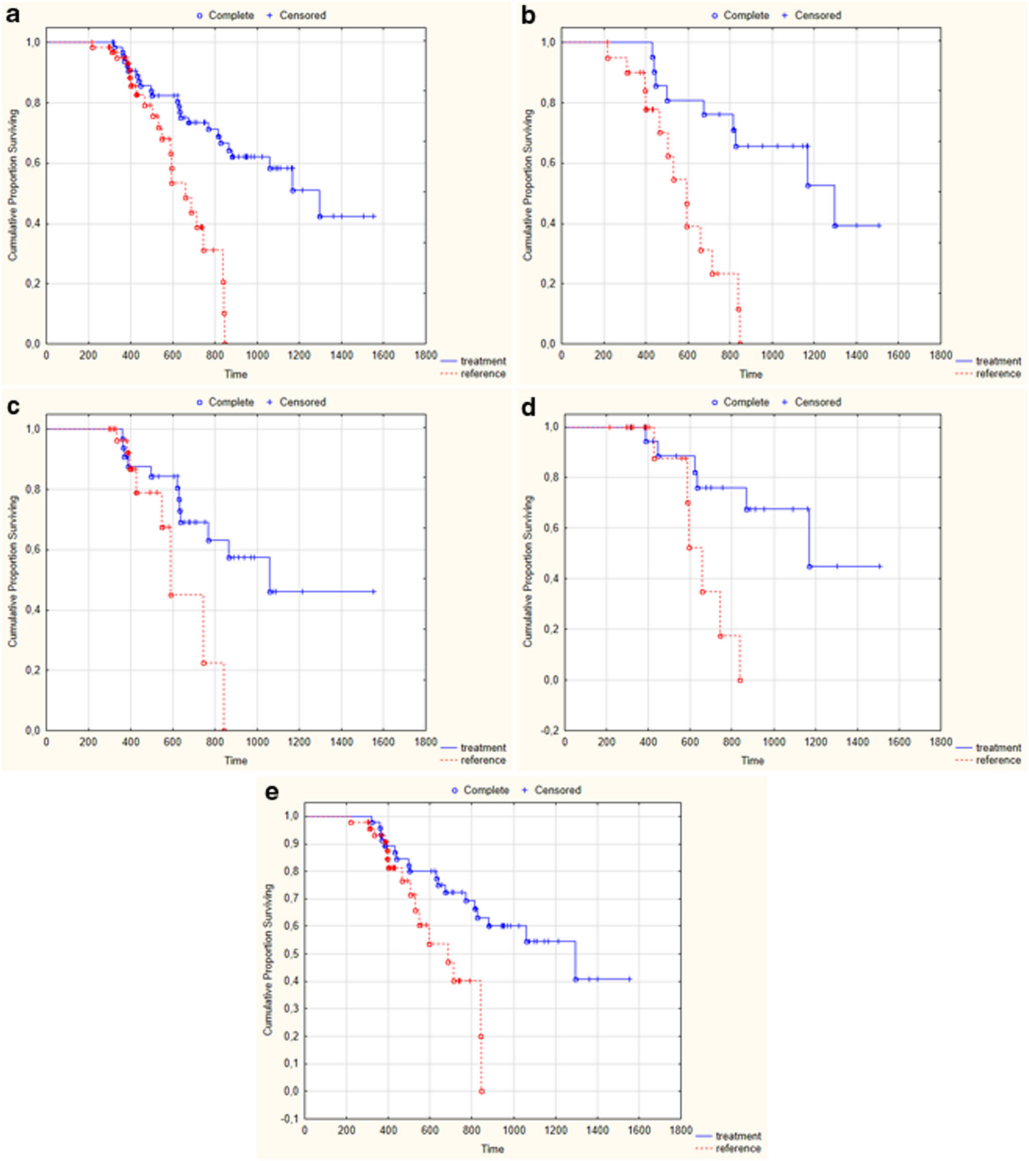
Overall survival in (**a**) the general study population, **b** patients with decreased progression rate, **c** patients with no change in progression rate, **d** more advanced patients, **e** less advanced patients

In the entire study population, the risk of death was decreased in patients treated with MSCs versus the paired control group by 70% (HR 0.30 [95% CI 0.16 to 0.59], *p* = 0.0004). Median overall survival was almost twice longer in MSC patients than in the reference group (1183 days vs. 640 days, *p* = 0.002). A similar effect on survival was observed in patients with lower disability at study entry (FT9, 1–2 stage) (*p* = 0.03), as well as for patients with higher disability at study entry (FT9, 3–5; p = 0.03) (Fig. 1). In the subgroup with decreased progression rate the difference was 1193 days vs. 568 days (*p* < 0.05 in all five tests) and in the subgroup with no change in progression rate it was 994 days vs. 580 days (p < 0.05 in the Cox-Mantel test). Kaplan-Meier curves for overall survival are presented in Fig. 1.

### MSCs Reduce ALS Progression Rates

**Fig. 2:**
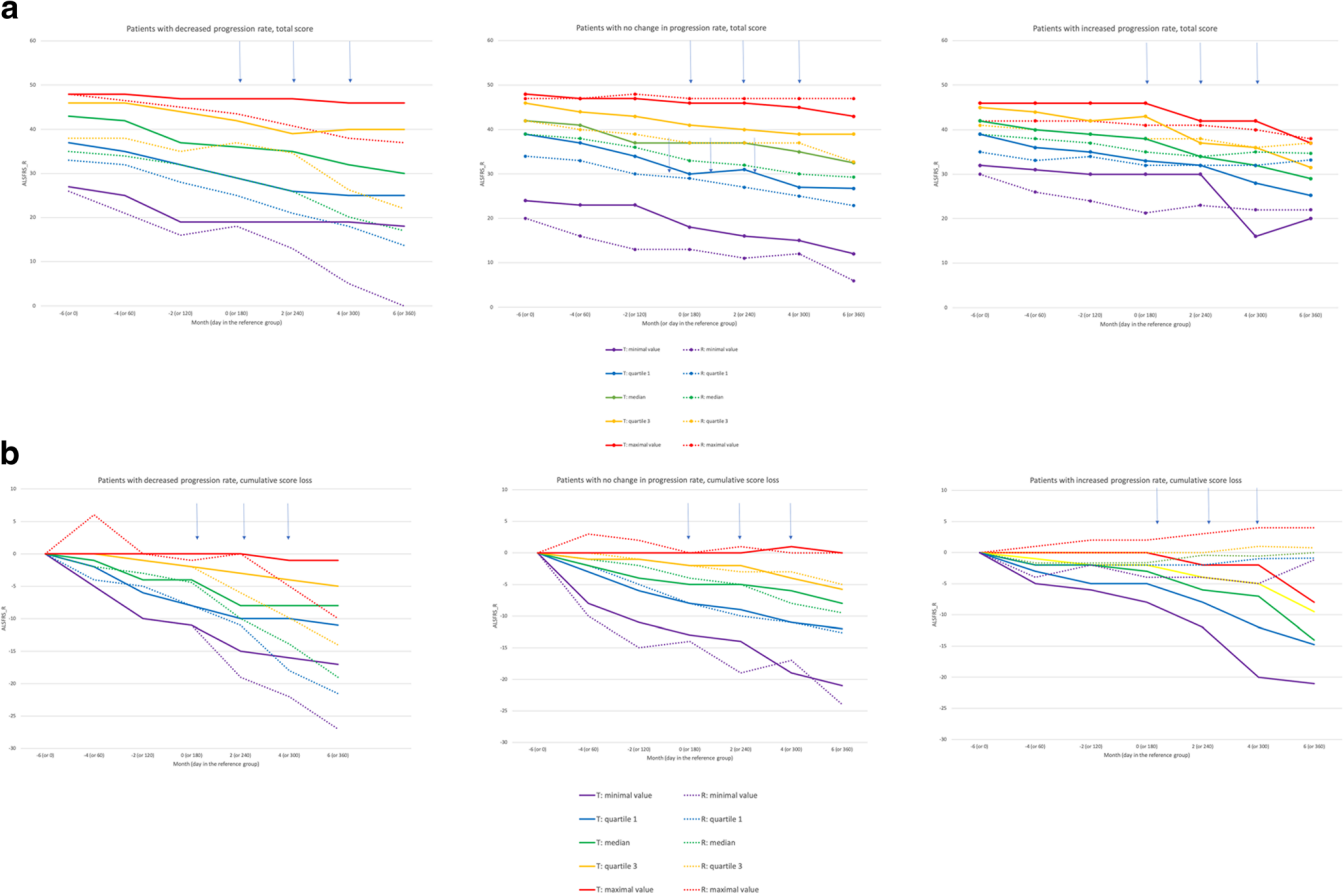
**a** ALSFRS-R total score in the subgroups divided by clinical response. **b**: ALSFRS-R cumulative change in the subgroups divided by clinical response. T - treatment group; R - reference group; MSC administrations are marked with arrows

In the whole study population, monthly score reduction (Fig. 2) during therapy was lower than prior to therapy (*p* = 0.008). From the total of 67 treated patients, 21 presented decrease in progression rate from the ALSFS-R evaluation vs. reference patients who progressed faster, 33 presented no difference in progression rate vs. reference patients, and 13 presented increase in progression rate when compared to the reference patients who progressed slower. These results were consistent with the linear regression analysis that revealed a significant (*p* = 0.0003) and strong (beta = −0.54, SE 0.15) effect of the MSC treatment. *A s*ubgroup analysis indicated that the risk-benefit ratio was favorable for patients who experienced decreased progression rate in all subgroups based on the initial patient characteristics (Table 2).

**Table 2:** Number and percent of patients with the individual response types in subgroups based on demographical and clinical predicting factors

*N* = 67	Decreased progression rate	No change in progression rate	Increased progression rate	Decreased or no change in progression rate outcome	No change or increased progression rate outcome	Risk-benefit ratio (increased to decreased progression rate response) ratio
General population	21	33	13	54	46	1: 1.6
N = 67	(31.3%)	(49.3%)	(19.4%)	(80.6%)	(68.7%)	
Sex						
Men (*n* = 37)	11 (29.7%)	16 (43.2%)	10 (27.1%)	27 (72.3%)	26 (70.3%)	1: 1.1
Women (*n* = 30)	10 (33.3%)	17 (56.7%)	3 (10.0%)	28 (93.3%)	20 (66.7%)	1: 5*
Age						
<median (*n* = 35)	14 (40.0%)	14 (40.0%)	7 (20.0%)	28 (80.0%)	21 (60%)	1: 2
≥median (*n* = 32)	7 (21.9%)	19 (59.4%)	6 (18.7%)	27 (84.4%)	25 (78.1%)	1: 1.4
Stage						
Less advanced (FT9 1–2) (n = 21)	5 (23.8%)	11 (52.4%)	5 (23.8%)	17 (81.0%)	16 (76.2%)	1: 1.25
More advanced (FT9 3–5) (*n* = 46)	16 (34.8%)	22 (47.8%)	8 (17.4%)	38 (82.6%)	30 (65.2%)	1: 2
Progression						
Fast (> = 1 point/month) (*n* = 26)	9 (34.6%)	14 (53.9%)	3 (11.5%)	23 (88.5%)	17 (65.4%)	1: 3
Slow (<1 point/ month) (*n* = 41)	12 (29.3%)	19 (46.3%)	10 (24.4%)	32 (78.0%)	29 (70.7%)	1: 1.3
Onset						
Bulbar (*n* = 16)	4 (25.0%)	10 (62.5%)	2 (12.5%)	15 (93.8%)	12 (75.0%)	1: 4
Spinal (*n* = 51)	17 (33.3%)	23 (45.1%)	11 (21.6%)	40 (78.4%)	34 (66.7%)	1: 1.5
Riluzole						
yes (*n* = 50)	17 (34.0%)	22 (44.0%)	11 (22.0%)	40 (80.0%)	33 (66.0%)	1: 1.7
no (*n* = 17)	4 (23.5%)	11 (64.7%)	2 (11.8%)	15 (88.2%)	13 (76.5%)	1: 2

*p < 0.05

### Early Response Predicts the Final Outcome of MSC Treatment

Discriminant analysis nor logistic regression allowed to identify no single demographic or clinical factor that would be a significant predictor for the type of response; the whole model consisting of these factors was also not significant. We found that following the first MSCs administration ALS patients with a better response in the ALSFRS-R versus reference patients also achieved a better final outcome, defined as a higher probability of having a better ALSFRS-R score after 6 months of MSC treatment. The estimate for effect exerted by the first dose response on the final outcome was at −0.33 (95% CI −0.58 to −0.1, *p* = 0.0059) in the logistic regression. The values for each ALSFRS-R score difference are presented in Table 3.

**Table 3:** Therapeutic failure probability depending on the difference between the result observed in a patient and the result obtained by the reference subject for this patient (early response). Data presented in the table below were obtained using logistic regression

Early response	Therapeutic failure probability
15	2.0%
14	2.8%
13	3.8%
12	5.3%
11	7.2%
10	9.9%
9	13.3%
8	17.6%
7	23.0%
6	29.5%
5	37.0%
4	45.1%
3	53.4%
2	61.6%
1	69.2%
0	75.9%
−1	81.5%
−2	86.0%
−3	89.6%
−4	92.3%
−5	94.4%
−6	95.9%
−7	97.1%
−8	97.9%
−9	98.5%
−10	98.9%
−11	99.2%
−12	99.4%
−13	99.6%
−14	99.7%
−15	99.8%

### Safety

None of the patients from the analyzed group treated with MSCs experienced any serious adverse events related to the cell administration. One patient presented signs of post lumbar puncture syndrome, such as nausea and headache within 1 day following the injection. Four patients suffered from the same syndrome one week following the injection. Two cases of a temperature above 37 degrees Celsius were also reported. Signs of the PLP syndrome were however short-lived and resolved without any complications. No new or unexpected reactions following the lumber puncture and MSCs administration were observed. During the two months following MSCs administration none of the patients reported any unexpected symptoms apart from their ALS. No meningeal signs were detected. No malignancies were reported in the follow-up period.

## Discussion

In her March 2020 JAMA Neurology publication, Vijayaraghavan highlighted the problem of limited access to clinical studies for patients with ALS. She described the case of her husband, a 38-years-old neuroscientist suffering from rapidly progressing ALS who – after being excluded from a clinical study – received stem cell therapy when it was already too late (due to the lengthy process of application for extended access) and died [19]. Without calling into the obvious objections related to unproven therapies based on stromal vascular fraction (SVF) [20, 21] or ill-defined “stem cells” [22] (http://ema.europa.eu/en/human-regulatory/overview/advanced-therapy-medicinal-products-overview, https://www.fda.gov/consumers/consumer-updates/fda-warns-about-stem-cell-therapies) administered by clinicians without the proper background or even by persons without a medical license [23], this case clearly shows that there are risks attached not only to action, but also to omission. Not every unregistered therapy should be considered unproven [24]. In the European Union, the Regulation 1394/2007 and national regulations passed under the 2001/83/EC Directive approve compassionate use of unregistered advanced therapy medicinal products manufactured under Good Manufacturing Practice. This is done as part of a Hospital Exemption subject to the approval of an ethics committee. Our results were obtained within this legal framework.

Our study yielded highly encouraging results when it comes to WJ-MSC administration in all subgroups distinguished based on demographical and clinical factors. For comparison, riluzole studies showed a survival slope change only in patients with the bulbar disease. This drug has been approved for ALS in 1994 [4], although it extends the life expectancy only by approximately 3 months. In 2017, a new medication, edaravone, was registered in the USA. We showed that WJ-MSC therapy extends survival to a comparable extent (HR 0.30 in our study vs. 0.33 for edaravone [25]). Our results confirm the previously published studies that reported on the safety and/or efficacy of MSC administration in patients with ALS in smaller groups [26–36], but we are the first to present a survival analysis in such a large patient population.

In 2019, the FDA [37] and a group of experts [38] published guidelines on the conduct of ALS clinical studies, with a goal of exploring its clinical and genetic biomarkers. Therefore, apart from reporting general efficacy, an important question, especially when considered that therapy was financed by the patients themselves or charities, regarded the prediction of response to MSC treatment. Our research indicated that the female sex and a positive clinical response (decreased progression rate) to the first MSC administration when compared to the strictly matched reference patient is a significant predictor for overall efficacy of the treatment. We would like to propose this tool for clinicians to allow them to obtain more informed consent, accordingly to the recent suggestions [39–41].

The results of this study are encouraging, however several limitations must be stressed. The case-control study is well established in the evidence-based hierarchy as better than case series, but weaker than randomized, double-blind, controlled studies. The case-control design assumes equality of the groups and the selection of a single control for each patient. Such selection may be fraught with a selection bias. In a group with 67 pairs we may expect this bias to affect all subgroups, but the reader must bear in mind that coincidence cannot be excluded; this refers to both patients who progressed slower and faster after administration. For this reason, a more beneficial avenue to be pursued for clinical use may be comparing the treated patient with the group of all reference subjects matching the criteria, which was not possible in this analysis due to the case-control design, which requires an equal number of patients. Secondly, the cut-off for the ALSFRS-R functional assessment follow-up period was 6 months following last administration. This was due to the fact that in the reference group the observation period rarely exceeded 12 months, out of which 6 months were used in this analysis as the comparison to the pre-treatment period. Assessment of the clinical response persistence in patients who experienced decreased progression rate was therefore based on linear regression modelling. Thirdly, we did not control genetic factors which may influence survival even in patients with sporadic ALS [42], thus we cannot exclude that the reason for the observed improvement is genetic mismatch, especially since as much as 50% of the variance in ALS has a genetic background [43].

Our study did not investigate the mechanisms involved in clinical improvement. The suggested role of natural killer cells [44] and regulatory T cells [45, 46] is in line with the mechanism of action of MSC described by other authors [47–50]. Some studies indicated that human neural stem cells transplanted into a spinal cord in patients with ALS differentiated into neurons which were still detected 2.5 years after administration; however, it is not known whether the same happens after administration of MSC [51]. Future studies should combine survival and molecular analyses. Suggested predictors include neutrophils and CD4 T cells [52], Serum Retinol-Binding Protein 4 [53], C-reactive protein [54] and 233 differential expressed genes in ALS monocytes, especially those related to inflammation (IL1B, IL8, FOSB, CXCL1, CXCL2) [55]. Nevertheless genetic testing is unfortunately still limited, even in clinical studies. Moreover, further studies into the factors related to donor selection, dosage and dosing schemes may be opportunities to achieve even better results.

Keeping in mind the study limitation, our results suggest that treatment with MSC substantially reduces the rate of progression and yields a twofold extension of survival in those who achieved a positive response to the first administration in comparison to the reference subject. These HR values were even better than those achieved with the registered drug, edaravone. Demographic factors have a certain impact on the risk-benefit ratio, so they should be discussed with the patient before the start of therapy. MSC administration was absolutely safe. Eligibility for the treatment should be confirmed following first administration based on the progression rate in comparison with at least one reference subject (more would be beneficial). Future studies should focus on confirming these observations in double-blind, controlled studies, optimally with a three-arm design to investigate additive or synergic effects with edaravone.

## Electronic supplementary material

Supplementary Fig. 1 Histogram of differences in age between patients treated with mesenchymal stem cells and the paired reference persons (PNG 75 kb)

High Resolution (TIF 61 kb)

Supplementary Fig. 2 Histogram of differences in the rate of progression between the patients treated with mesenchymal stem cells and the paired reference persons (PNG 205 kb)

High Resolution (TIF 63 kb)

Supplementary Fig. 3 Histogram of differences in the rate of progression between the patients being treated and the paired reference person. The power of survival analysis depending on the size of the treated group (PNG 297 kb)

High Resolution (TIF 856 kb)
