# Supplementary figures and images for: Umbilical Cord Mesenchymal Stem Cells in Amyotrophic Lateral Sclerosis: an Original Study
Source: Stem Cell Rev Rep. 2020 Jul 28;16(5):922–32. doi: 10.1007/s12015-020-10016-7 (PMC7456414; doi:10.1007/s12015-020-10016-7)

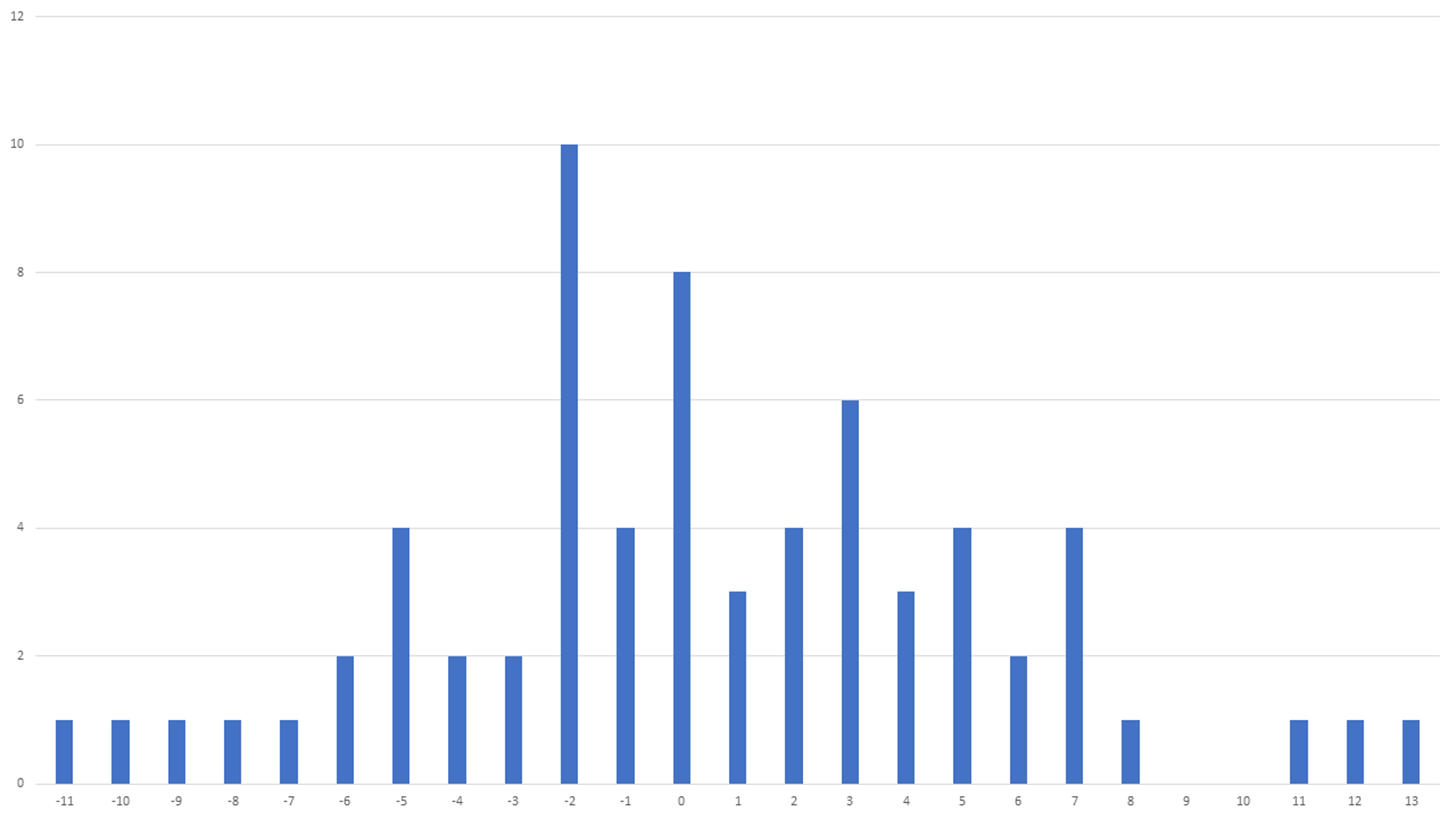

Supplement: Supplementary file 1 — Histogram of differences in age between patients treated with mesenchymal stem cells and the paired reference persons (PNG 75 kb) [file 12015_2020_10016_Fig3_ESM.png]

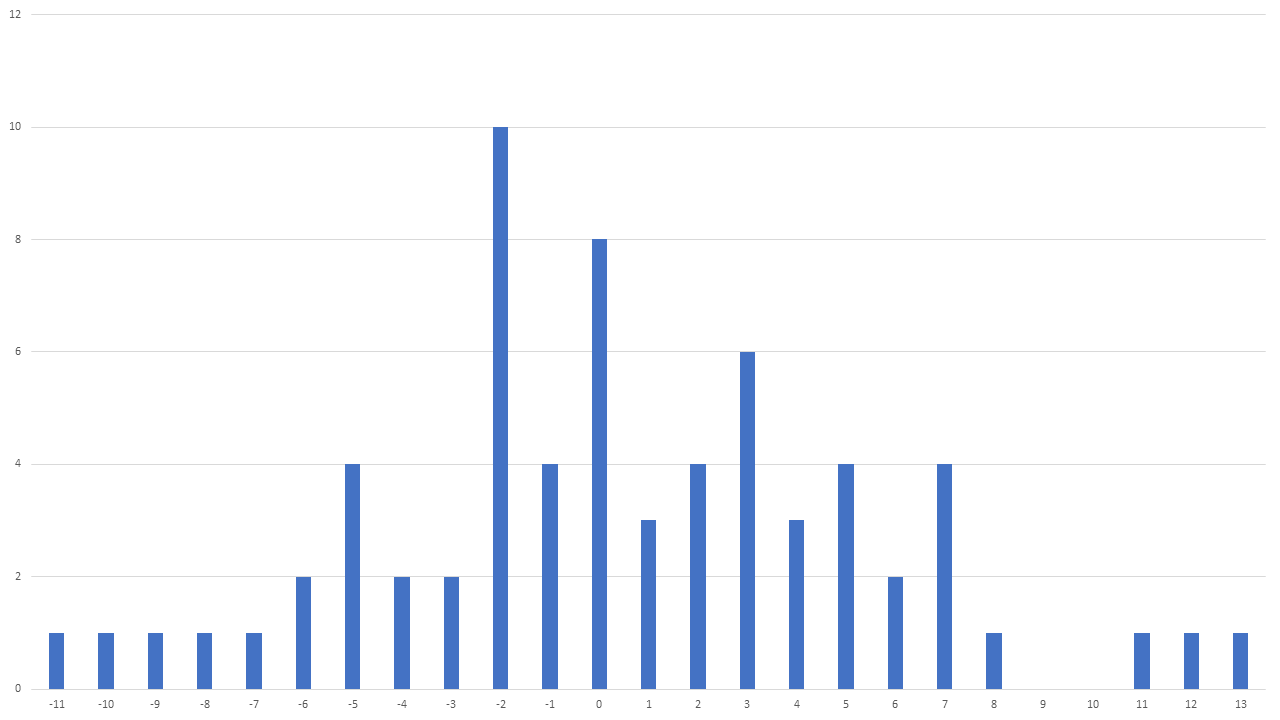

Supplement: Supplementary file 2 — High Resolution (TIF 61 kb) [file 12015_2020_10016_MOESM1_ESM.tif]

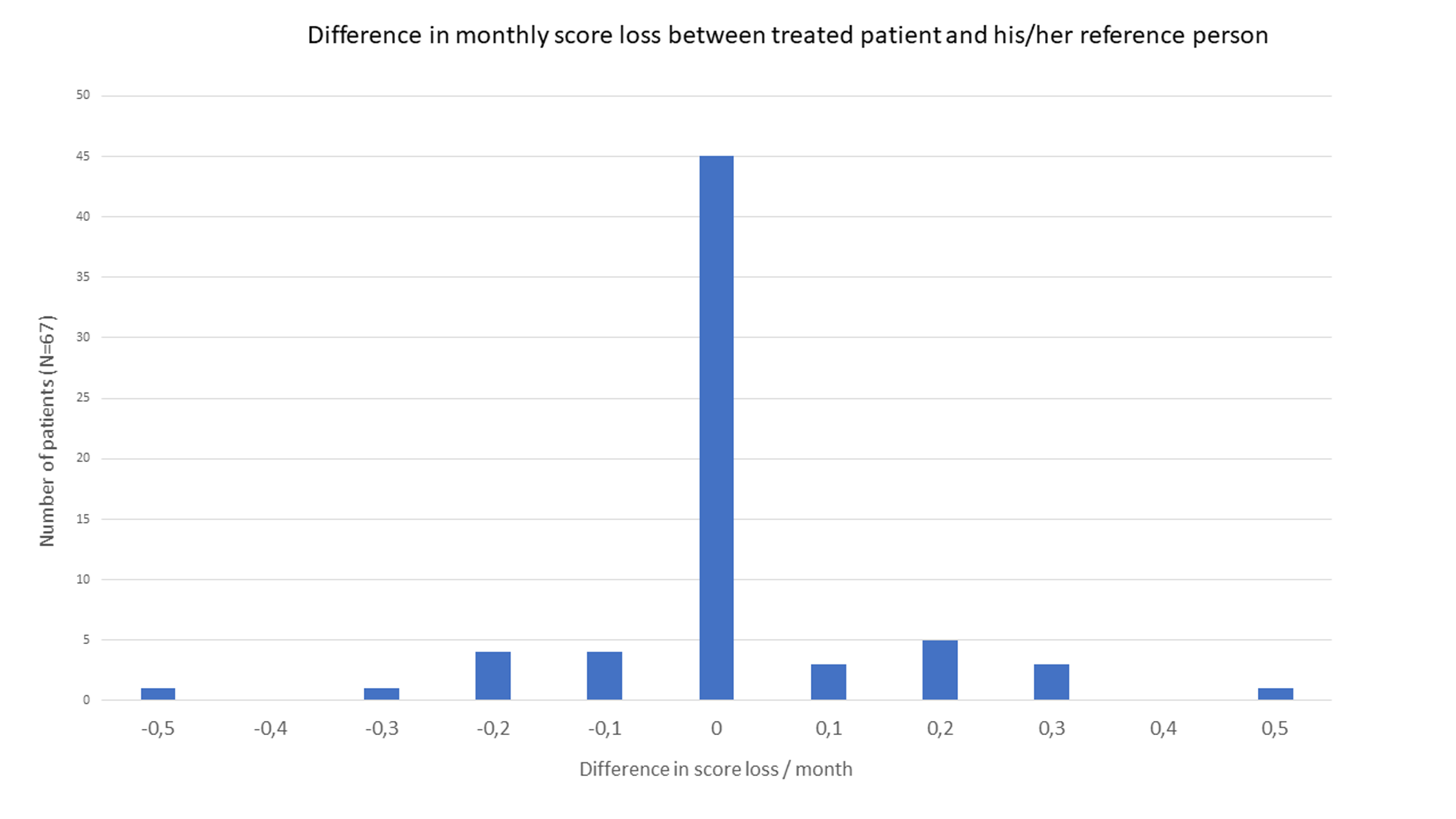

Supplement: Supplementary file 3 — Histogram of differences in the rate of progression between the patients treated with mesenchymal stem cells and the paired reference persons (PNG 205 kb) [file 12015_2020_10016_Fig4_ESM.png]

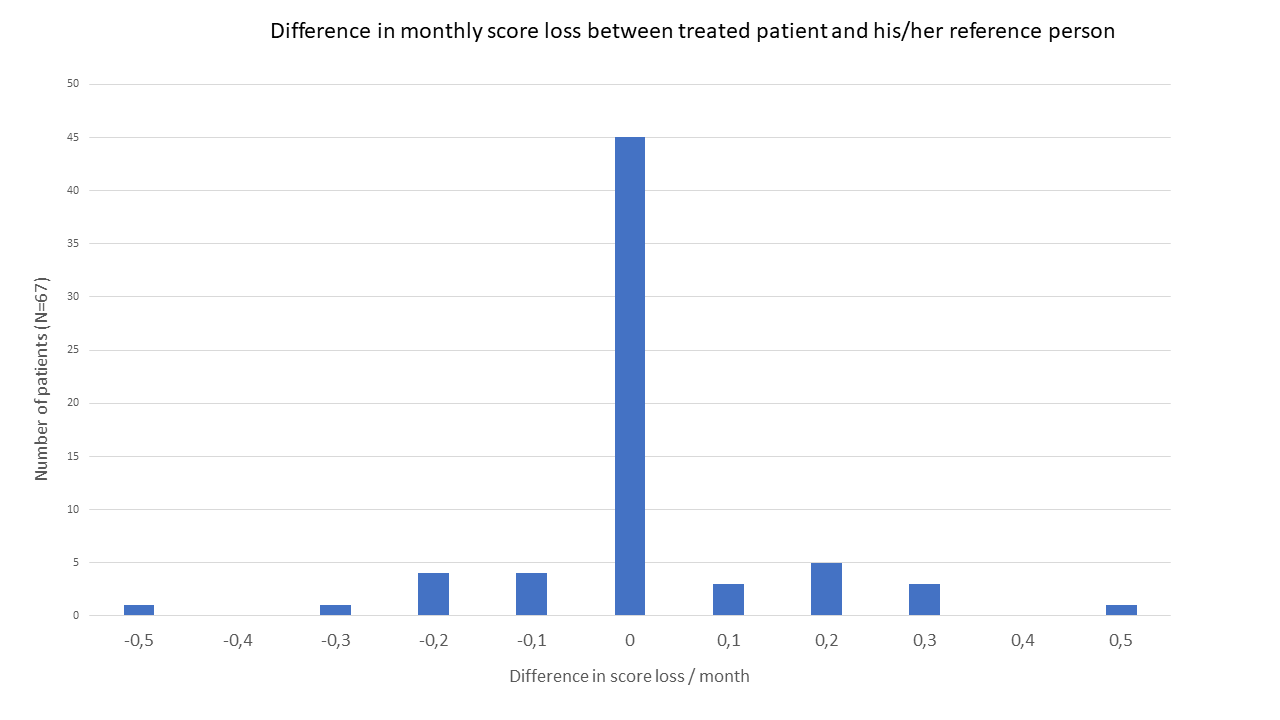

Supplement: Supplementary file 4 — High Resolution (TIF 63 kb) [file 12015_2020_10016_MOESM2_ESM.tif]

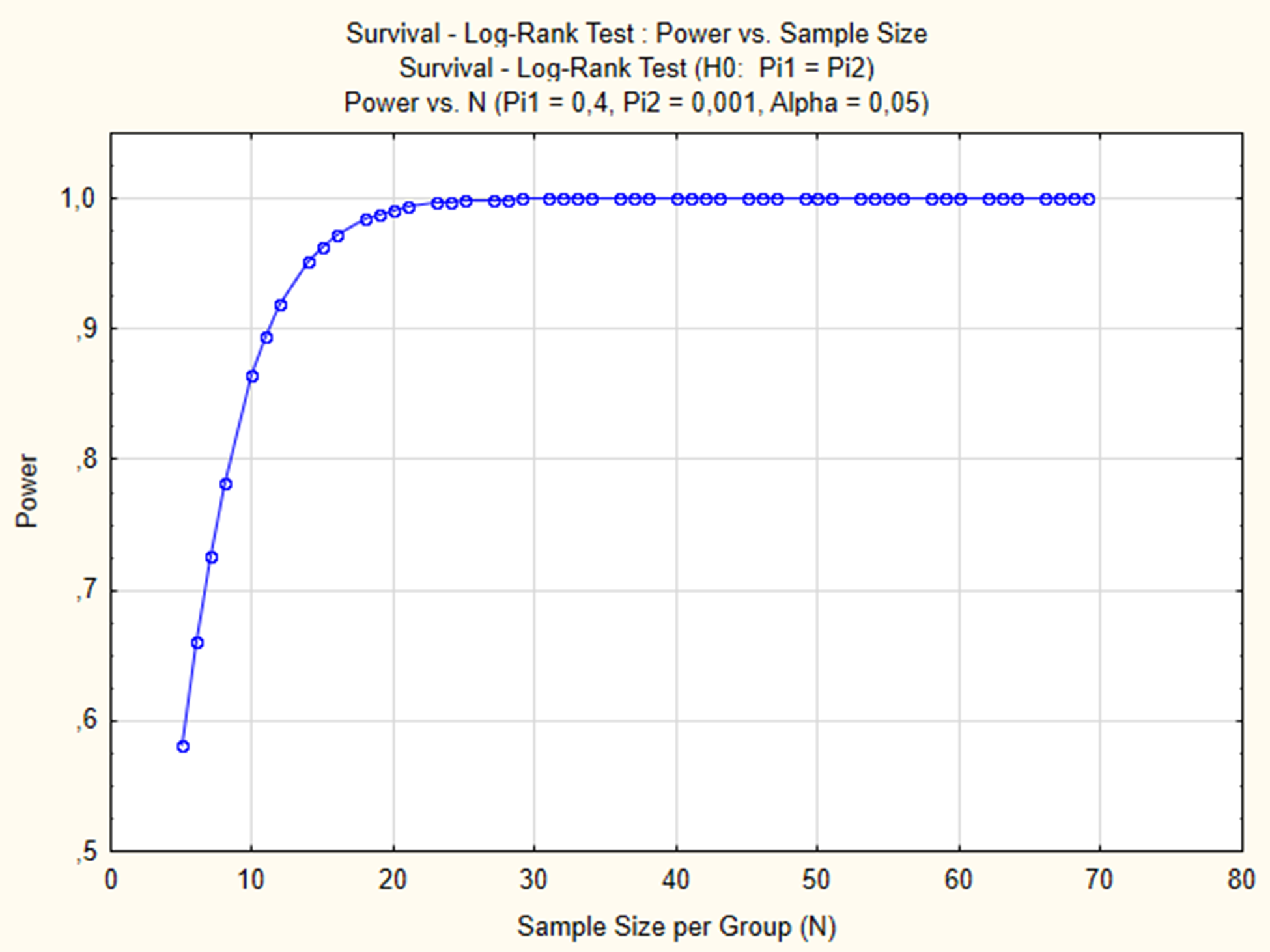

Supplement: Supplementary file 5 — Histogram of differences in the rate of progression between the patients being treated and the paired reference person. The power of survival analysis depending on the size of the treated group (PNG 297 kb) [file 12015_2020_10016_Fig5_ESM.png]

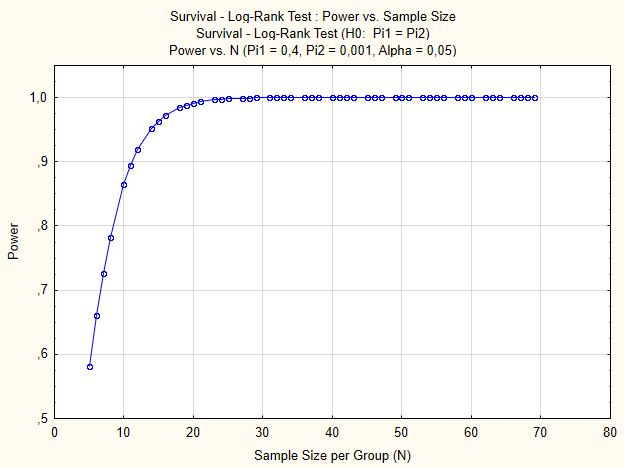

Supplement: Supplementary file 6 — High Resolution (TIF 856 kb) [file 12015_2020_10016_MOESM3_ESM.tif]
